# Point mutation of tyrosine 759 of the IL-6 family cytokine receptor, gp130, augments collagen-induced arthritis in DBA/1J mice

**DOI:** 10.1186/1471-2474-10-23

**Published:** 2009-02-19

**Authors:** Fumio Tsuji, Miwa Yoshimi, Osamu Katsuta, Miwa Takai, Katsuhiko Ishihara, Hiroyuki Aono

**Affiliations:** 1 Research and Development Center, Santen Pharmaceutical Co., Ltd., 8916-16 Takayama-cho, Ikoma-shi, Nara 630-0101, Japan; 2 Department of Immunology and Molecular Medicine, Kawasaki Medical School, Kurashiki, Okayama 701-0192, Japan

## Abstract

**Background:**

Knock-in mice (gp130F759) with a Y759F point mutation in gp130, a signal transducing receptor subunit shared by members of the IL-6 cytokine family, show sustained activation of STAT3, enhanced acute-phase or immune responses, and autoimmune arthritis. We conducted a detailed analysis of collagen-induced arthritis (CIA) in gp130F759 with a DBA/1J background (D/J.gp130F759).

**Methods:**

We backcrossed gp130F759 to C57BL/6 and DBA/1J, and compared the pathologic changes, including occurrence of arthritis, in the two distinct genetic backgrounds. We analyzed CIA in D/J.gp130F759 and investigated the effects of methotrexate (MTX) on CIA.

**Results:**

C57BL/6 background gp130F759 mice, but not D/J.gp130F759, spontaneously developed polyarthritis and glomerulonephritis. On the other hand, keratitis of the eyes only developed in D/J.gp130F759, indicating the influence of genetic background on disease development in gp130F759 mice. Resistance of the DBA/1J background against spontaneous arthritis urged us to examine CIA in D/J.gp130F759. CIA in D/J.gp130F759 was more severe, with greater bone destruction, than the control mice. After collagen immunization, splenomegaly and serum levels of rheumatoid factor and anti-DNA antibody were augmented in D/J.gp130F759. Bio-Plex analysis of serum cytokines revealed increased IL-12p40 and PDGF-BB before immunization, and increased levels of IFN-γ, IL-17, TNF-α, IL-9, and MIP-1β 8 days after the booster dose. IL-6 and PDGF-BB in D/J.gp130F759 showed distinct kinetics from the other cytokines; higher levels were observed after arthritis development. MTX partially attenuated the development of arthritis and inhibited bone destruction in D/J.gp130F759, with reduction of anti-type II collagen antibody levels, suggesting that MTX mainly affects antigen-specific immune responses in CIA.

**Conclusion:**

The Tyr-759 point mutation of the IL-6 family cytokine receptor subunit, gp130, caused autoimmune disease, and this was also influenced by the genetic background. CIA in D/J.gp130F759 is useful for evaluating drugs in a relatively short period because sustained activation of STAT3 may enhance the disease symptoms.

## Background

Rheumatoid arthritis (RA) is a systemic autoimmune disease characterized by progressive chronic inflammation of multiple joints, resulting in joint destruction. Several characteristics of RA, such as hyper-γ-globulinemia, autoantibody production, genetic linkage with the HLA-DR locus, and infiltration of T-cells and plasma cells into the synovium, have suggested that immunologic disorders have crucial roles in the pathogenesis of this disease [1]. RA is a polygenic disease caused by immunologic disorders that develop from the synergistic actions of genetic and environmental factors. Clinical and experimental studies have revealed the pivotal roles for inflammatory cytokines, such as tumor necrosis factor (TNF)-α, interleukin (IL)-1, and IL-6, in the pathophysiology of RA [2]. Success achieved in the blockade of TNF-α in RA and IL-6 in juvenile RA exemplifies the feasibility and potential therapeutic application of antagonizing cytokine signaling [3].

IL-6 is a pleiotropic cytokine that regulates various biologic functions, such as the development of the nervous and hematopoietic systems, acute-phase responses, inflammation, and immune responses [4]. A causative role for IL-6 in autoimmune disease was first recognized by the observation that the autoimmune symptoms of patients with cardiac myxomas, such as hyper-γ-globulinemia and autoantibody production, disappeared with resection of the tumor that produced IL-6 [5]. Furthermore, a high concentration of IL-6 exists in the synovial fluid and sera of RA patients [6]. Studies using IL-6 knockout mice revealed that IL-6 is involved in the severity and progress of experimentally-induced arthritis [7-9]. Clinical trials of a humanized anti-IL-6Rα monoclonal antibody (MRA) have provided evidence supporting the crucial roles for IL-6 in RA pathophysiology [3].

The IL-6 receptor comprises two molecules, IL-6 receptor α-chain and gp130, which is shared among the receptors for the IL-6 cytokine family. Ligand binding of gp130 activates two major signal-transduction pathways (the STAT3-mediated signal and the SHP-2/Gab/MAPK signal) in a manner dependent on the YXXQ motif and tyrosine (Y) 759 of gp130, respectively [10,11].

To clarify the roles of SHP-2- and STAT3-mediated signal-transduction pathways *in vivo*, we generated a series of knock-in mouse lines in which the gp130-mediated STAT3 or SHP-2 signals were selectively disrupted. This was achieved by mutating the tyrosine residues of all the YXXQ motifs or Y759 to phenylalanine (*gp130*^*FXXQ*/*FXXQ *^and *gp130*^*F*759/*F*759 ^mice, respectively [the latter is hereafter abbreviated gp130F759]). Analyses of these mice indicated that SHP-2-mediated or Y759-dependent signals negatively regulate the biological responses elicited by the STAT3-mediated signals *in vivo*, and that the balance of positive and negative signals generated through gp130 is skewed or shifted to positive STAT3 signaling in gp130F759 [12]. Importantly, gp130F759 in the mixed background with 129 and C57BL/6 spontaneously develop a RA-like autoimmune disease in old age [13]. gp130F759 shows severe immunologic abnormalities, including autoantibody production, increased memory/activated T-cells, impaired thymic negative selection, and peripheral clonal deletion. Development of RA-like disease is entirely dependent on mature lymphocytes, but abnormally enhanced homeostatic proliferation of CD4 T-cells is caused by augmented production of IL-7 by non-hematopoietic stromal cells through a STAT3-dependent process [14]. However, the 129/C57BL/6 mixed background gp130F759 mice were not suitable for drug evaluation due to the late onset (approximately 1 year). We, therefore, backcrossed gp130F759 to DBA/1J, which is known to develop arthritis by immunization of heterologous type II collagen, with the expectation of an earlier development of spontaneous arthritis.

Here, we report the histopathologic abnormalities in two genetic backgrounds of gp130F759, and analyze collagen-induced arthritis (CIA) in gp130F759 with a DBA/1J background for the development of a model for drug evaluation.

## Methods

### Animals

Experimental procedures were conducted with the approval of the Santen Animal Experimental Ethics Committee.

*gp130*^*F*759/*F*759 ^knock-in mice were backcrossed to C57BL/6 or DBA/1J eight times (Japan SLC Inc., Hamamatsu, Japan), hereafter abbreviated B6.gp130F759 and D/J.gp130F759, respectively. In all studies, we used wild-type mice (Japan SLC Inc.) as controls. Mice were housed in a specific pathogen-free animal facility at the Research and Development Center of Santen Pharmaceutical Company Limited. A 12-h light/dark cycle was maintained in a room at 23 ± 1°C. Mice had free access to food and water.

### Organ analysis

Mice were sacrificed at 12 months for the comparative study of mice with two genetic backgrounds. Eyes, limbs, spleen, thymus, mesenteric lymph nodes, lungs, and kidneys were histologically-examined. The weights of the spleen and thymus were recorded. For the study of CIA, mice were sacrificed at the end of the experimental period (day 56) by exsanguination. After measuring the weights of the spleen and thymus, the organs were fixed in 10% neutral buffered formalin solution. After fixation, the joints were decalcified in 10% ethylenediaminetetraacetate solution for 3 weeks. Paraffin-embedded samples were sectioned and stained with hematoxylin and eosin. Sections were examined under light microscopy.

### CIA

CIA was induced in D/J.gp130F759 and wild-type mice according to the method described previously [15]. We previously reported that arthritis in D/J.gp130F759 mice was more severe than in control mice when the interval immunization of bovine type II collagen was 3 weeks [16]. In this study, we chose an interval immunization protocol of 2 weeks to develop arthritis in a shorter period. Mice were injected intradermally into the base of the tail with 200 μg of bovine type II collagen emulsified in Freund's complete adjuvant (FCA). Two weeks after the initial injection, a booster injection of 200 μg of bovine type II collagen emulsified in FCA was administered intradermally into the base of the tail. The activity of clinical arthritis was evaluated twice a week from the first immunization (day 0) until day 56. The severity of clinical arthritis in the limbs was scored as 0 (no arthritis), 1 (swelling of one digit), 2 (swelling of two or more digits), 3 (swelling of the paw), and 4 (swelling of the paw and changes in the bone). The arthritic score was the sum of the scores of all involved joints. The joints were processed for histologic analysis at the end of the experimental period (56 days). Proliferation of synovial cells, joint destruction, and lymphocyte infiltration into the synovial lining were blindly evaluated. The histopathologic scores of the hind paw were graded on the basis of the changes to the intact joints, as described previously [17]: 0 (negative), 1 (very slight: slight change in a few joints, no change in many joints), 2 (slight: moderate change in few joints, no change or slight change in many joints), 3 (moderate: severe or moderate change in many joints, slight change in a few joints), and 4 (severe: severe change in every joint).

For drug evaluations, methotrexate (MTX; Sigma, St. Louis, MO, USA) at 0.2 mg/kg was administered p.o. once daily from the first immunization until day 42, and the activity of clinical arthritis was evaluated. At the end of the experimental period (42 days), the joints were processed for histologic examination. Proliferation of synovial cells, joint destruction, and lymphocyte infiltration into the synovial lining were blindly evaluated.

### IL-2 production in lymph node cells

Mice were sacrificed 14 days after the first immunization. The inguinal lymph nodes were removed, and single-cell suspensions were prepared in complete medium (RPMI1640 supplemented with 1% autologous mouse serum, 5 × 10^-5 ^M 2-mercaptoethanol, 100 units/mL penicillin, and 100 μg/mL streptomycin). Lymph node cells (4 × 10^6 ^cells/mL) with 200 μL of complete medium were cultured in the presence or absence of 5 μg/mL of bovine type II collagen or 1 μg/mL of concanavalin-A for 2 days. Thereafter, supernatants were collected for measurement of IL-2 production.

### Detection of cytokines, autoantibodies, and cartilage oligomeric matrix protein (COMP)

Serum cytokines were measured using the Bio-Plex suspension array system (Biorad, Hercules, CA, USA). Mouse IL-7, rheumatoid factor (RF) for mouse immunoglobulin (Ig) of IgM classes, and anti-ssDNA antibodies were measured using a commercial ELISA kit (R&D Systems, Minneapolis, MN, USA; Shibayagi, Gunma, Japan). Levels of anti-type II collagen antibody were measured by ELISA using a mouse IgG anti-type II collagen antibody assay kit (Chondrex, Redmond, WA, USA). COMP levels were measured using a commercial ELISA kit (AnaMar Medical, Göteborg, Sweden).

### Statistical analyses

Results were evaluated by Student's *t*-test (EXSAS; Arm, Osaka, Japan).

## Results

### Abnormalities in gp130F759 knock-in mice with distinct genetic backgrounds

Abnormalities in gp130F759 mice with the C57BL/6 or DBA/1J background are shown in Tables 1 &2. The characteristic abnormalities of B6.gp130F759 mice in both sexes were polyarthritis and hyperplasia of the spleen and lymph node. Glomerulonephritis only developed in females. These abnormalities were not observed in the DBA/1J background. D/J.gp130F759 mice were resistant to spontaneous polyarthritis, even though DBA/1J-background mice were known to develop CIA. Cellular infiltration in the lung and hypertrophy of the thymus were observed in both backgrounds. On the other hand, keratitis developed with a much higher prevalence in the DBA/1J background. These results indicated that the pathologic changes caused by the Y759F point mutation of gp130 were affected by the genetic background of the mice.

**Table 1:** Histopathological analysis of gp130F759 mice (12 months) Male

	C57BL/6		DBA/1J	
Findings	Wild	F759	Wild	F759

Joint (lower extremity); Synovial cell proliferation	0/5	5/5	0/5	0/5

Kidney; Glomerulonephritis	0/5	0/5	0/5	0/5

Eye; Keratitis	0/5	0/5	1/5	4/5

Lung; Cellular infiltration	0/5	5/5	0/5	3/5

Spleen; Hyperplasia, plasma cell	0/5	5/5	0/5	0/5

Thymus; Hypertrophy/hyperplasia, medulla	0/5	5/5	0/5	2/5

Lymph node (mesenteric); Hyperplasia, plasma cell	0/5	4/5	0/5	0/5

**Table 2:** Histopathological analysis of gp130F759 mice (12 months) Female

	C57BL/6		DBA/1J	
Findings	Wild	F759	Wild	F759

Joint (lower extremity); Synovial cell proliferation	0/5	5/5	2/5	0/5

Kidney; Glomerulonephritis	0/5	2/5	0/5	0/5

Eye; Keratitis	0/5	1/5	0/5	3/5

Lung; Cellular infiltration	3/5	3/5	1/5	1/5

Spleen; Hyperplasia, plasma cell	0/5	5/5	0/5	0/5

Thymus; Hypertrophy/hyperplasia, medulla	0/5	5/5	0/5	4/5

Lymph node (mesenteric); Hyperplasia, plasma cell	2/5	5/5	0/5	0/5

### CIA in DBA/1J background gp130F759 mice

**Figure 1:**
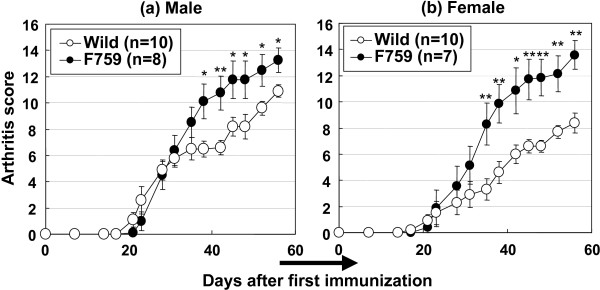
**Severe arthritis developed in gp130F759 mice by collagen immunization**. D/J.gp130F759 and wild-type mice were injected intradermally with collagen emulsified in FCA on day 0 and day 14. Values are expressed as mean ± standard error of 7 to 10 animals. *P < 0.05, **P < 0.01 versus wild-type mice by Student's t-test.

We attempted to induce CIA in DBA/1J background knock-in mice to determine if the point mutation affected the prevalence or course of CIA because D/J.gp130F759 mice did not develop spontaneous arthritis up to 4 months of observation (data not shown). The severity of polyarthritis in D/J.gp130F759 mice was higher than that in wild-type mice (Fig. 1). The prevalence of polyarthritis at day 56 was 100% in D/J.gp130F759 and wild-type mice. The degree of arthritis in gp130F759 mice was more severe in the hind limbs than in the forelimbs. Therefore, we examined the histologic changes in the hind limbs, such as proliferation of the synovial cells, joint destruction, and lymphocyte infiltration into the synovial lining (Table 3). The histologic analysis showed that the severity of arthritis was greater with high bone destruction in gp130F759 mice than in wild-type mice. The histopathologic grades of gp130F759 mice tended to converge at 2 or 3, whereas those of wild-type mice diverged from 0–4.

**Table 3:** Destructive arthritis developed in CIA in DBA/1J gp130F759 mice

Day 56		Male		Female	
		Wlid	F759	Wild	F759
	Grade	20*	16*	20*	14*
Proliferation of the synovial lining cells	0	7	0	8	1
	1	1	1	4	0
	2	3	3	2	1
	3	7	10	4	11
	4	2	2	2	1

Destruction of the articular cartilage/bone	0	9	3	14	1
	1	0	0	1	0
	2	4	0	2	0
	3	7	8	2	13
	4	0	5	1	0

Inflammatory cell infiltration, rich in neutrophils	0	10	3	12	1
	1	1	0	2	1
	2	1	11	2	6
	3	5	2	3	5
	4	3	0	1	1

All data refer to the number of limbs with the finding in the group.

*Total number of limbs in the group

Grade of change: 0, negative; 1, very slight; 2, slight; 3, moderate; 4, severe.

**Figure 2:**
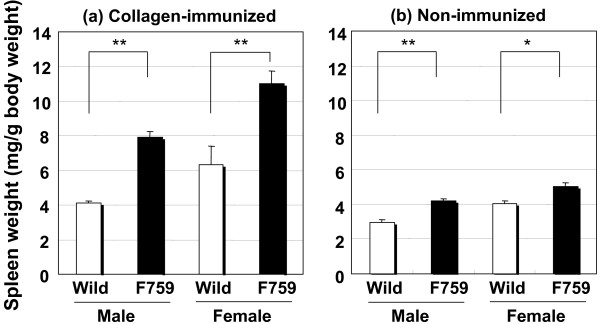
**Marked splenomegaly in CIA of D/J.gp130F759 mice**. Values are expressed as mean ± standard error of 7 to 10 animals of collagen-immunized mice on day 56 (a) and 3 to 4 animals of non-immunized mice (b). *P < 0.05, **P < 0.01 versus wild-type mice by Student's t-test.

**Figure 3:**
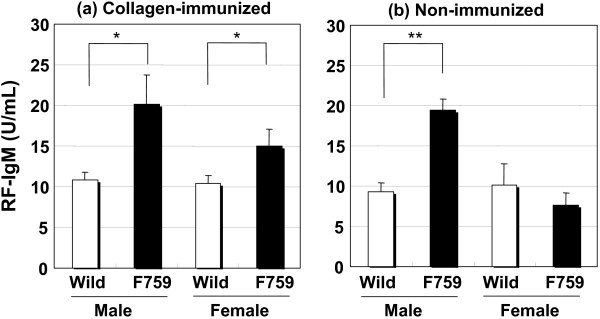
**Increased serum levels of rheumatoid factor in CIA of D/J.gp130F759 mice**. Values are expressed as mean ± standard error of 7 to 10 animals of collagen-immunized mice on day 56 (a) and 3 to 4 animals of non-immunized mice (b). *P < 0.05, **P < 0.01 versus wild-type mice by Student's t-test.

**Figure 4:**
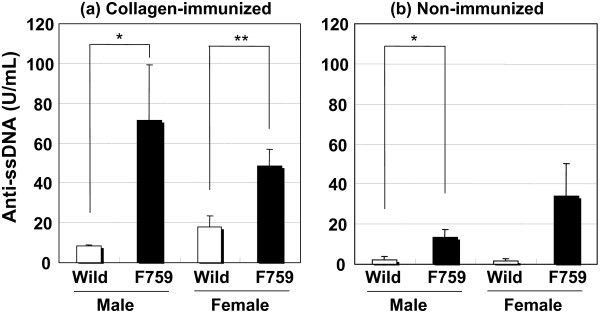
**Increased serum levels of anti-ssDNA antibody in CIA of D/J.gp130F759 mice**. Values are expressed as mean ± standard error of 7 to 10 animals of collagen-immunized mice on day 56 (a) and 3 to 4 animals of non-immunized mice (b). *P < 0.05, **P < 0.01 versus wild-type mice by Student's t-test.

**Figure 5:**
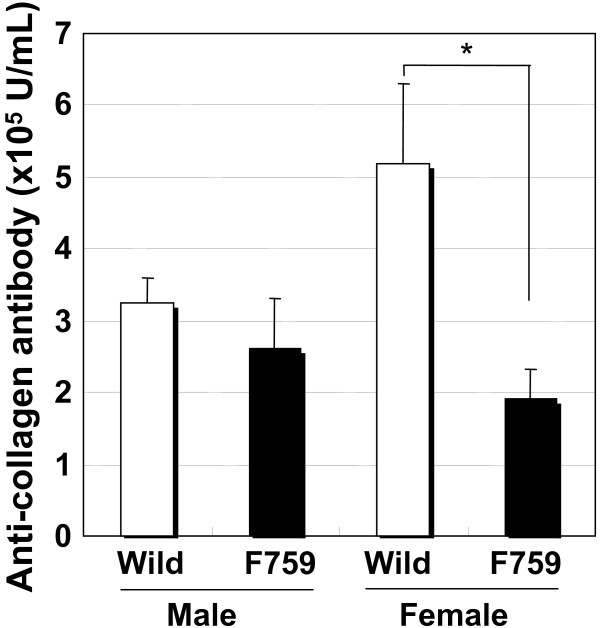
**Serum levels of anti-collagen antibody in CIA of D/J.gp130F759 mice on day 56**. Values are expressed as mean ± standard error of 7 to 10 animals on day 56. *P < 0.05 versus wild-type mice by Student's t-test.

Before immunization, spleen weights in D/J.gp130F759 mice were approximately 1.3-times higher than the control mice. After immunization, the weights increased approximately 1.8-times compared to the controls (Fig. 2). Before immunization, the serum level of RF was a little higher in gp130F759 mice with little differences between males and females, which did not change significantly by collagen immunization (Fig. 3). The serum level of anti-ssDNA antibody was higher in gp130F759 mice before and after immunization (Fig. 4). The level of anti-type II collagen antibody, which was scarcely detected before immunization, was lower in gp130F759 mice, even after immunization, with little differences between males and females (Fig. 5).

**Figure 6:**
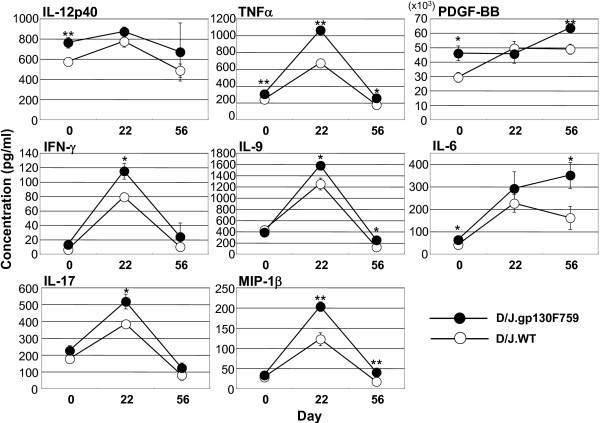
**Serum cytokines that showed different responses in D/J.gp130F759 mice (female)**. Serum cytokine levels were measured by Bio-Plex system. Values are expressed as mean ± standard error of 7 to 10 animals. *P < 0.05, **P < 0.01 versus wild-type mice at same day by Student's t-test.

Bio-Plex analysis of serum cytokines revealed that non-stimulated female D/J.gp130F759 mice showed significantly increased levels of IL-12(p40) and platelet-derived growth factor-BB (PDGF-BB; Fig. 6). On day 22, 8 days after the second immunization, the serum levels of TNF-α, interferon (IFN)-γ, IL-17, IL-9, and macrophage inflammatory protein (MIP)-1β were significantly higher in female D/J.gp130F759 mice, all of which returned to basal levels comparable with those of wild-type mice on day 56. Levels of IL-6 and PDGF-BB on day 56 further increased in female D/J.gp130F759 mice, whereas levels declined or were unchanged in wild-type mice. Serum IL-7 was not detectable in CIA mice by ELISA (sensitivity, 10 pg/mL). On days 0 and 56, cytokine data in male and female mice were almost identical (data not shown). We did not conduct the cytokine analysis of male serum on day 22.

### IL-2 production in lymph node cells

**Figure 7:**
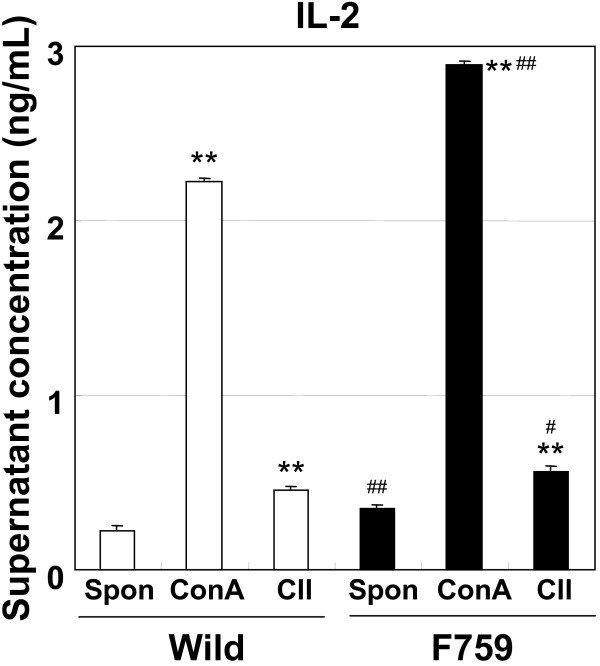
**IL-2 production in lymph node cells of D/J.gp130F759 mice (female) by collagen and concanavalin A stimulation**. Values are expressed as mean ± standard error of 12 samples. **P < 0.01 versus non-treated group (Spon) by Student's t-test. #*P *< 0.05, ## *P *< 0.01 versus same condition group of wild type mice-derived lymph node cells by Student's t-test.

IL-2 production in lymph node cells of female D/J.gp130F759 mice stimulated by collagen and concanavalin-A was slightly higher than in control mice (Fig. 7). In addition, spontaneous IL-2 production in lymph node cells of D/J.gp130F759 mice was also slightly higher than in the control mice. Lymphocyte proliferation in gp130F759 mice and the control mice was similar in both backgrounds (data not shown). These results suggest that the Y759F mutation of gp130 scarcely affected T-cell immune responses in CIA.

### Effects of MTX on CIA in DBA/1J background gp130^*F*759/*F*759 ^knock-in mice

**Figure 8:**
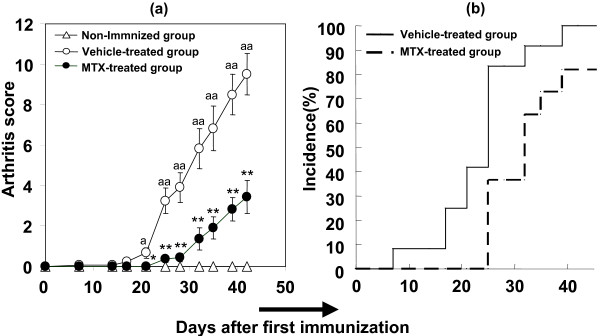
**Methotrexate attenuated CIA (a), and delayed the onset of arthritis (b) in gp130F759 mice (male)**. Values are expressed as mean ± standard error of 11 to 12 animals. *P < 0.05, **P < 0.01 versus vehicle-treated group by Student's t-test. ^a^*P *< 0.05, ^aa^*P *< 0.01 versus Non-immunized group by Student's t-test.

**Figure 9:**
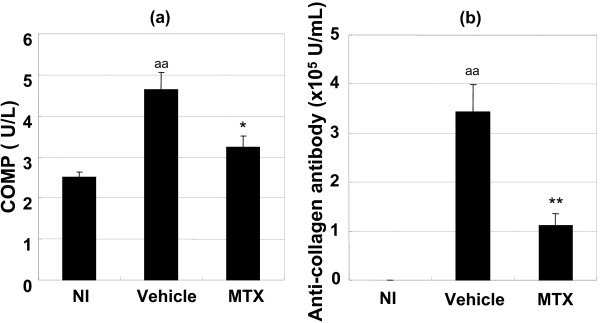
**Methotrexate attenuated the increase of COMP levels (a) and anti-collagen antibody levels (b) in the serum on day 42**. Values are expressed as mean ± standard error of 11 to 12 animals. *P < 0.05, **P < 0.01 versus vehicle-treated group by Student's t-test. ^aa^*P *< 0.01 versus Non-immunized group (NI) by Student's t-test.

We investigated the effects of MTX on CIA in DBA/1J background male gp130F759 mice to determine if this model would be useful for drug evaluation. Severe polyarthritis was observed in the vehicle-treated group, but MTX (0.2 mg/kg/day) partially attenuated it (Fig. 8a). MTX also delayed the onset of polyarthritis (Fig. 8b). Histologic analysis showed that severe arthritis with high bone destruction was observed in the vehicle-treated group, which was inhibited by MTX (Table 4). The COMP data also supported these findings in the histopathologic analysis. The increased serum level of COMP in the vehicle-treated group at the end of the experimental period was nearly suppressed by treatment with MTX (Fig. 9a). MTX also attenuated the anti-type II collagen antibody level, which was increased by collagen immunization (Fig. 9b). Despite histologic improvement, MTX scarcely affected the serum level of anti-ssDNA antibody, RF, MIP-1β, TNF-α, IL-6, IL-9, IL-17, and IFN-γ at the end of the experimental period (data not shown).

**Table 4:** Methotrexate attenuated CIA in gp130F759 mice (male, histopathological analysis)

Day 42		Vehicle	MTX
	Grade	24*	22*
Proliferation of the synovial lining cells	0	7	16
	1	0	0
	2	12	3
	3	5	3
	4	0	0

Destruction of the articular cartilage/bone	0	6	16
	1	0	0
	2	3	2
	3	15	4
	4	0	0

Inflammatory cell infiltration, rich in neutrophils	0	5	15
	1	0	0
	2	4	1
	3	11	6
	4	4	0

All data refer to the number of limbs with the finding in the group.

*Total number of limbs in the group

Grade of change: 0, negative; 1, very slight; 2, slight; 3, moderate; 4, severe.

## Discussion

Many abnormalities were shown in B6.gp130F759 mice at 12 months of age. Characteristic abnormalities in C57BL/6 background knock-in mice were not only spontaneous polyarthritis, but also glomerulonephritis (only in females). Spontaneous arthritis did not develop in the DBA/1J background, indicating that the genetic background of DBA/1J is resistant to spontaneous arthritis caused by the Y759F mutation of gp130. On the other hand, keratitis was characteristic in D/J.gp130F759 mice. These different abnormalities between mice with two backgrounds may have been caused by differences in their H-2 haplotype (C57BL/6; b, DBA/1J; q). Thymic hyperplasia was observed in both backgrounds, but splenic hyperplasia only occurred in the C57BL/6 background. This abnormality may be an important requisite for the development of spontaneous arthritis.

We tried to induce CIA in DBA/1J background knock-in mice resistant to spontaneous polyarthritis. The severity of polyarthritis in gp130F759 mice was greater in wild-type mice when the 2-week interval immunization protocol was followed, which was similar to our previous study results involving a 3-week interval immunization protocol [16]. In the 2-week interval immunization protocol, the development of arthritis was observed more rapidly than in the 3-week interval immunization protocol. To achieve a score of 10, it takes almost 50 days from the first immunization by the 3-week interval immunization protocol [16], but it takes only 40 days by the 2-week interval immunization protocol. The severity of arthritis in gp130F759 mice was greater than in the control mice. From cytokine analysis, the high level of IL-6 in serum was maintained in gp130F759 mice after the second immunization. Serum levels of IL-6 are also correlated with clinical indices of disease activity in RA [18], and high levels of IL-6 may trigger a positive-feedback loop of gp130 signaling. Serum levels of PDGF-BB, one of the angiogenesis factors produced mainly by macrophages, was also elevated. It has been reported that PDGF and the PDGF receptor are overexpressed in RA synovial tissue, and PDGF is a potent stimulator of synovial hyperplasia in RA [19-21]. The tyrosine kinase inhibitor, imatinib, attenuates PDGF receptor signaling in fibroblast-like synoviocytes, and potently prevents and treats murine CIA [22]. PDGF-BB induces IL-6 production in osteoblasts [23]. These findings suggest that a high level of PDGF-BB in the serum of gp130F759 mice may be involved in angiogenesis and synovial hyperplasia. Further studies to clarify the mechanisms for sustained elevation of IL-6 and PDGF-BB need to be conducted in the future.

In gp130F759 mice with CIA, the serum levels of TNF-α, IL-9, IL-17, IFN-γ, and MIP-1β were elevated 8 days after the second immunization. TNF-α has an important role in RA [2], particularly growth of synovial fibroblasts. IFN-γ has long been known to be a critical T-helper (Th)1 cytokine in the initiation and perpetuation of inflammation and autoimmune disease [24]. It has been reported that anti-IFN-γ-treated RA patients demonstrate significant clinical improvement [25]. IL-9 is considered to a Th2 cytokine, and has been shown to act on many cell types, including T cells, B cells, mast cells, eosinophils, and neutrophils [26]. IL-9 relates to survival of T cells and antibody production from B cells, and also induces IL-6 secretion from bone marrow-derived mast cells. However, the pathologic role of IL-9 in RA is unknown.

Elevation of IL-17 in gp130F759 mice [16] may be produced by IL-17-producing helper T-cells (Th17). STAT3, activated by IL-6 and IL-23, has a critical role in Th17 development [16,27]. CIA is reported to be a Th17-dependent autoimmune disease [28]. The development of Th17 in gp130F759 mice may, therefore, contribute to augmentation of the inflammatory response in CIA.

MIP-1β is a member of the CC chemokine subfamily, and has been shown to signal mainly through CCR5 [29]. It has been demonstrated that monocytes produce high amounts of MIP-1β when stimulated with IL-7 [30]. We reported that autoimmune disease in gp130 mutant mice is caused by increased homeostatic proliferation of CD4^+ ^T-cells, which is due to elevated production of IL-7 by non-hematopoietic cells as a result of IL-6 family cytokine-gp130-STAT3 signaling [14]. The high level of MIP-1β may therefore be due to elevated production of IL-7 in the micro-environment, although it could not be detected in the serum by ELISA. It has been reported that MIP-1β expression in RA synovial fluid is elevated, and MIP-1β may participate in the selective recruitment of CCR5^+^CXCR3^+ ^T-cells to the inflamed synovium [31]. Bao et al. [32] reported that CCR5 knockout mice show a significant reduction in the prevalence of CIA after collagen-immunization as compared with wild-type mice. Elevated MIP-1β in gp130F759 mice may partially contribute to arthritis.

RF and anti-DNA antibody levels were higher in gp130F759 mice, but anti-type II collagen antibody level was slightly lower. IL-2 production in lymph node cells of collagen-immunized D/J.gp130F759 mice stimulated by collagen was slightly higher than that of the control mice. These results suggest that immune reactions in gp130F759 mice and the control mice against heterologous collagen have slightly different properties. However, antigen-specific immune responses were not as highly augmented in gp130F759 from the present data. Therefore, the augmentation of arthritis in gp130F759 mice may be due to the increased response of inflammation or innate immunity. Further studies to clarify the relation between the augmentation of arthritis in gp130F759 mice and the humoral or cellular immunity need to be conducted in the future.

We investigated the effects of MTX, which is widely used for RA treatment, on this model. It has been reported that MTX ameliorates T-cell-dependent autoimmune arthritis (including CIA), but not antibody- or fibroblast-induced arthritis [33]. In CIA in D/J.gp130F759 mice, MTX attenuated arthritis development and inhibited bone destruction. MTX also attenuated elevation of anti-type II collagen antibody. Svensson et al. [34] reported that B-cell-deficient mice did not develop CIA. Attenuation of anti-type II collagen antibody level may, therefore, be one of the mechanisms for the efficacy of MTX on CIA in D/J.gp130F759 mice. It is intriguing that MTX attenuated arthritis with reduction of anti-type II collagen antibody, which is not enhanced in D/J.gp130F759 mice. We could not detect significant decreases in the serum level of anti-ssDNA antibody, RF, MIP-1β, TNF-α, IL-6, IL-9, IL-17, and IFN-γ in the MTX-treated group at the end of the experimental period, but all factors tended to decrease in the MTX-treated group. It has been reported that MTX modulates cytokine production by T-cells and macrophages in murine CIA [35]. The immunosuppressive mechanism of MTX by cytokine modulation may be important for its clinical usefulness. We therefore consider that CIA in D/J.gp130F759 mice in a 2-week interval immunization protocol is useful for drug evaluation in a relatively short period because sustained activation of STAT3 may enhance the disease symptoms. Recently, JAK1/2 inhibitor acting on IL-6-induced STAT3 phosphorylation demonstrated clinical activity in RA [36]. Genetic factors contribute to the anti-inflammatory efficacy of MTX, and DBA/1J mice are resistant to MTX [37]. Further studies may be needed to clarify the similarity between CIA in D/J.gp130F759 and RA.

## Conclusion

The point mutation of Tyr-759 of the IL-6 family cytokine receptor subunit, gp130, causes autoimmune disease, and this is also influenced by the genetic background. CIA in D/J.gp130F759 is useful for evaluating drugs in a relatively short period because sustained activation of STAT3 may enhance the disease symptoms.

## Abbreviations

B6.gp130F759: C57BL/6 background gp130F759; CCR5: chemokine (C-C motif) receptor 5; CIA: collagen-induced arthritis; COMP: cartilage oligomeric matrix protein; D/J.gp130F759: DBA/1J background gp130F759; ELISA: enzyme-linked immunosorbent assay; FCA: Freund's complete adjuvant; Gab: GRB2-associated binding protein; IFN: interferon; IL: interleukin; MAPK: mitogen-activated protein kinase; MIP-1β: macrophage inflammatory protein-1 beta; MTX: methotrexate; PDGF: platelet-derived growth factor; RA: rheumatoid arthritis; RF: rheumatoid factor; SHP-2: Src homology 2-conteining tyrosine phosphatase; STAT3: signal transducer and activator of transcription 3; Th: T helper; TH17: IL-17-producing helper T cells; TNF-α: tumor necrosis factor alpha

## Competing interests

The authors declare that they have no competing interests.

## Authors' contributions

FT carried out animal experiments, cell culture experiments, and cytokine and antibody detection. FT also performed the statistical analysis, and wrote the manuscript. MY and OK carried out the histologic studies. MT performed animal experiments. KI gave valuable advice to FT. HA supervised the study design. All authors approved the final manuscript.

## Pre-publication history

The pre-publication history for this paper can be accessed here:


